# The survival strength of younger patients in BCLC stage 0-B of hepatocellular carcinoma: basing on competing risk model

**DOI:** 10.1186/s12885-022-09293-x

**Published:** 2022-02-18

**Authors:** Huiwen Yan, Xinhui Wang, Xiaoli Liu, Peng Wang, Lihua Yu, Dongdong Zhou, Zhiyun Yang

**Affiliations:** 1 Center of Integrative Medicine, Capital Medical University Affiliated Beijing Ditan Hospital, No. 8 Jing Shun East Street, Beijing, 100015 People’s Republic of China; 2 Dongzhimen Hospital, Beijing University of Chinese Medicine, Chaoyang District, Beijing, 100029 People’s Republic of China

**Keywords:** Hepatocellular carcinoma, Younger, Barcelona clinic liver Cancer classification, Prognosis, Survival

## Abstract

**Background:**

The number of young patients with hepatocellular carcinoma (HCC) is increasing, but whether patients of different ages have a survival advantage is unclear. This study was conducted to investigate whether age differences in the Barcelona Clinic Liver Cancer (BCLC) classification system contribute to the long-term survival outcomes of patients with HCC.

**Methods:**

A total of 1602 patients with HCC admitted to the Beijing Ditan Hospital was included in this study. Patients were divided into younger (≤45 years) and older (> 45 years) groups. Factors determining overall survival and progression-free survival were analyzed using univariate and multivariate analyses with the Kaplan-Meier method and Cox proportional hazard regression model. We calculated the cumulative incidence function using the Fine-Gray model. The effect of mortality on age was also estimated using a restricted cubic spline.

**Results:**

After matching, overall survival and progression-free survival were significantly better in younger patients than in older patients with BCLC stage 0-B (*p* = 0.015 and *p* = 0.017, respectively). In BCLC stage 0-B, all-cause mortality increased with age and increased rapidly around the age of 40 years (non-linear, *p* < 0.05). In BCLC stages 0-B, HCC-related and non-HCC-related deaths significantly differed between younger and older individuals (*p* = 0.0019).

**Conclusion:**

In stage BCLC 0-B, age affects the long-term prognosis of patients.

**Supplementary Information:**

The online version contains supplementary material available at 10.1186/s12885-022-09293-x.

## Introduction

Hepatocellular carcinoma (HCC), a malignant lesion of the liver cells, is a major diseases that seriously endangers human life and health, and is a major public health issue. More than 500,000 new cases of HCC are diagnosed globally each year [1, 2]. Hepatitis B virus (HBV) and hepatitis C virus (HCV) infection are the two most important causes of HCC, with HBV associated with HCC in 60–70% of patients in Asian countries, and HCV infection in 60–80% of patients in western countries. Liver cancer mainly occurs in older populations (> 45 years of age) with a higher incidence of comorbidities, which are typically considered as high-risk groups for treatment [3]. The incidence of liver cancer in younger patients is increasing with improvements in liver cancer screening among high-risk groups [4, 5]. Several studies showed that the prognosis of elderly patients with HCC is worse than that of younger patients, whereas other studies reported a worse prognosis in younger patients or no difference in prognosis between younger and older patients [6–9].

The Barcelona Clinical Liver Cancer (BCLC) staging system provides better predictive value for early diagnosis. Most patients are initially diagnosed with mid- to late-stage liver cancer in a vague manner [10, 11]. As a result, the BCLC staging system has been widely used for the diagnosis and evaluation of HCC.

These contradictory results may be related to comparisons between different age groups, regardless of the tumor staging. We compared the survival outcomes of HCC in older (> 45 years) and younger (≤45 years) patients according to BCLC staging.

## Materials and methods

### Diagnosis and staging of HCC

We conducted a retrospective cohort study of 1602 patients with HCC using data from the Beijing Ditan Hospital from January 2009 to December 2018. HCC was diagnosed based on the recommendations of the European Association for the Study of Liver Diseases and the American Association for the Study of Liver Diseases (AASLD). The inclusion criteria were as follows: (1) clear clinical diagnosis of HCC and (2) patients with complete clinical data. Exclusion criteria included: (1) autoimmune liver disease; hepatitis A, D, or E; syphilis; acquired immunodeficiency syndrome; or other primary malignancy; (2) metastatic liver cancer; (3) pregnant women; and (4) patients with unclear BCLC staging. The primary outcome was the death of patients with HCC, with the observation time defined as the time between the patient’s first participation in this observational study and date of death. Patient survival was defined as the time between the diagnosis of liver cancer and death or the end of the study.

### Definition of BCLC stages

The BCLC staging system contains four main categories of prognostic factors: the patient’s general state, tumor state, liver function state, and available treatment options (2018 AASLD) [12]. The very early stage (0) is defined as solitary HCC (< 2 cm), early stage (A) is solitary or 2–3 nodules (≤3 cm), intermediate stage (B) is multinodular and unresectable, advanced stage (C) is the presence of portal invasion or extrahepatic spread, and terminal stage (D) is a severe physical condition (Performance Status (PS) score of 3–4) and end-stage liver function. We compared the demographic data and clinical factors between the two groups (BCLC stage 0-B and BCLC stage C-D).

### Demographics and clinical data

We extracted the following data for the study: sex, age, family history of HCC, alcohol abuse, esophageal and/or gastric varices, HBsAg, HCV, cirrhosis, portal vein tumor thrombus (PVTT), Child-Pugh score, tumor multiplicity, tumor size, white blood cell (WBC), platelet counts (PLT), alanine aminotransferase (ALT) and treatment at baseline. Laboratory values at the time of diagnosis of HCC are classified as elevated when they are higher than the clinical normal values. Tumor staging was performed using the BCLC staging method and liver function was assessed using the Child-Pugh scoring method.

### Statistical analysis

Demographics and clinical factors were compared between the two groups (age > 45 and ≤ 45 years groups), with categorical variables using the chi-square test. Overall survival (OS) and progression-free survival (PFS) were estimated using the Kaplan-Meier method and compared using the log-rank test. OS was defined as the period from the first diagnosis to death, independent of the date of the last follow-up. PFS was defined as the time between the first diagnosis and date of diagnosis of tumor progression or last follow-up. Cox proportional regression was used to determine age factors independently associated with BCLC stage 0-B. To assess prognostic factors independent of age, variables identified as significant in univariate analysis were included in the multivariate Cox proportional hazards model.

We further assessed the pattern of association between age and primary outcome using a Cox proportional hazards regression model with restricted cubic splines (RCS) for age, with 6 knots at 30, 40, 50, 60, 70, and 80 years of age. To determine the effect of age on HCC-related and non-HCC-related causes of death, we calculated a cumulative incidence function using the Fine-Gray model. HCC-related deaths included death from HCC and/or rupture of intra-abdominal liver cancer. Non-HCC death occurred because of a condition other than those mentioned above.

Statistical significance was set at *p* < 0.05. All statistical analyses were performed using R version 3.6.3 (The R Foundation for Statistical Computing, Vienna, Austria).

## Results

### Clinical characteristics of the study participants

Table 1 shows the demographic and clinical characteristics of the two groups based on age. The cohort included 1395 patients with HCC aged > 45 years and 207 patients with HCC aged ≤45 years. Prior to PS matching, the younger group of patients contained more men and showed a lower proportion of cirrhosis compared to the older patient group (Table 1) (*p* < 0.001). In addition, younger patients had a higher proportion of WBC, PLT and ALT. Because of the heterogeneity between younger and older patients at baseline, PS matching was performed for sex, BCLC staging, and three treatment types. Thereafter, younger and older patients were essentially identical in terms of tumor characteristics and type of treatment (Table S1).

**Table 1:** Demographic and clinical characteristics of study patients

Demographic and clinical values	Total (***N*** = 1602)	Age group > 45y (***N*** = 1395)	Age group ≤ 45y (***N*** = 207)	***P*** value
**Patient characteristics**				
**Sex**				< 0.001
Male	1254 (78.3)	1071 (76.8)	183 (88.4)	
Female	348 (21.7)	324 (23.2)	24 (11.6)	
**Alcohol abuse**				0.812
No alcohol	991 (61.9)	865 (62.0)	126 (60.9)	
Alcohol	611 (38.1)	530 (38.0)	81 (39.1)	
**Family history of HCC**				0.207
No	1545 (96.4)	1349 (96.7)	196 (94.7)	
Yes	57 (3.6)	46 (3.3)	11 (5.3)	
**HBsAg**				0.001
Negative	172 (10.7)	164 (11.8)	8 (3.9)	
Positive	1430 (89.3)	1231 (88.2)	199 (96.1)	
**HCV**				0.006
Negative	1532 (95.6)	1326 (95.1)	206 (99.5)	
Positive	70 (4.4)	69 (4.9)	1 (0.5)	
**Esophageal and/or gastric varices**				0.667
No	1231 (76.8)	1069 (76.6)	162 (78.3)	
Yes	371 (23.2)	326 (23.4)	45 (21.7)	
**Cirrhosis**				< 0.001
No	113 (7.1)	80 (5.7)	33 (15.9)	
Yes	1489 (92.9)	1315 (94.3)	174 (84.1)	
**PVTT at baseline**				0.031
No	1264 (78.9)	1113 (79.8)	151 (72.9)	
Yes	338 (21.1)	282 (20.2)	56 (27.1)	
**Child-Pugh Staging**				0.003
A	784 (48.9)	660 (47.3)	124 (59.9)	
B	589 (36.8)	531 (38.1)	58 (28.0)	
C	229 (14.3)	204 (14.6)	25 (12.1)	
**Tumor characteristics**				
**Tumor multiplicity**				0.306
Solitary	903 (56.4)	779 (55.8)	124 (59.9)	
Multiple	699 (43.6)	616 (44.2)	83 (40.1)	
**Tumor size**				0.186
≤5 cm	1097 (68.5)	964 (69.1)	133 (64.3)	
> 5 cm	505 (31.5)	431 (30.9)	74 (35.7)	
**BCLC staging**				0.257
0-B	1058 (66.0)	929 (66.6)	129 (62.3)	
C-D	544 (34.0)	466 (33.4)	78 (37.7)	
**Preoperative laboratory tests**				
**WBC (10^9/L)**				< 0.001
Low≤4	673 (42.0)	614 (44.0)	59 (28.5)	
High> 4	929 (58.0)	781 (56.0)	148 (71.5)	
**PLT(10^9/L)**				< 0.001
Low≤100	903 (56.4)	814 (58.4)	89 (43.0)	
High> 100	699 (43.6)	581 (41.6)	118 (57.0)	
**ALT (U/L)**				0.002
Low≤50	1166 (72.8)	1034 (74.1)	132 (63.8)	
High> 50	436 (27.2)	361 (25.9)	75 (36.2)	
**Type of treatment**				
**Resection**				0.072
No	1547 (96.6)	1352 (96.9)	195 (94.2)	
Yes	55 (3.4)	43 (3.1)	12 (5.8)	
**Palliative**				1
No	1199 (74.8)	1044 (74.8)	155 (74.9)	
Yes	403 (25.2)	351 (25.2)	52 (25.1)	
**Minimally invasive**				0.324
No	536 (33.5)	460 (33.0)	76 (36.7)	
Yes	1066 (66.5)	935 (67.0)	131 (63.3)	

*p* value between > 45y and ≤ 45y age groups

*PVTT* portal vein tumor thrombus, *WBC* white blood cell, *PLT* platelet, *ALT* alanine aminotransferase

### Survival analysis

**Fig. 1:**
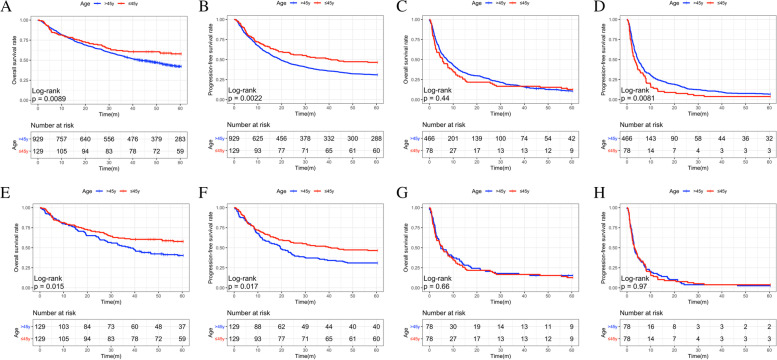
The Kaplan-Meier survival curves of overall survival (OS) and progression-free survival (PFS) in different BCLC stage HCC patients before and after PSM. **A**,**B** The OS (A) and PFS (**B**) in BCLC stage 0-B before PSM. **C**,**D** The OS (**C**) and PFS (**D**) in BCLC stage C-D before PSM. **E**,**F** The OS (**E**) and PFS (**F**) in BCLC stage 0-B after PSM. **G**,**H** The OS (**H**) and PFS (**H**) in BCLC stage C-D after PSM. HCC, hepatocellular carcinoma; BCLC, Barcelona Clinic Liver Cancer; PSM, Propensity Score Matching

Age was an independent predictor of survival in BCLC stages 0-B before (Fig. 1A-D) and after PS matching (Fig. 1E-H). Young patients showed better 5-year OS and PFS compared to older patients (*p* = 0.015 and *p* = 0.017, respectively; Fig. 1E, F). However, in BCLC C-D, there were no significant differences in OS and PFS between younger and older patients (*p* = 0.66 and *p* = 0.97, respectively; Fig. 1G, H).

### RCS of patient death versus age for different BCLC stages

**Fig. 2:**
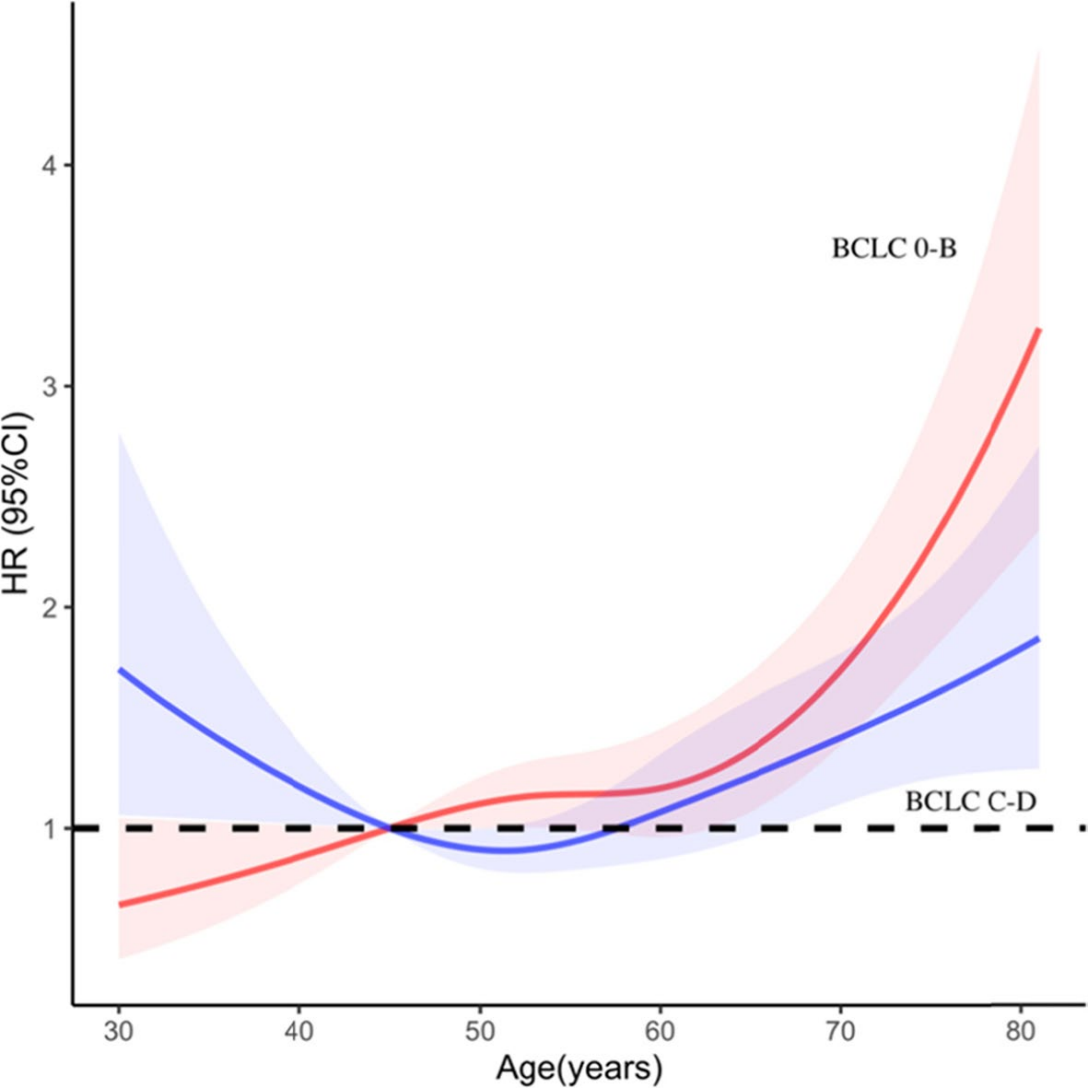
Age-varying effects on all-cause mortality in BCLC stage 0-B and BCLC stage C-D. HR and 95% confidence intervals were derived from a Cox proportional hazards regression model with restricted cubic splines, with 6 knots at 30, 40, 50, 60, 70, and 80 years of age. Reference age was 45 years. The red line and red area indicated HR and 95% CI of BCLC stage 0-B, and the blue line and blue area indicated HR and 95% CI of BCLC stage C-D. HR, hazard ratios; CI, confidence interval; BCLC, Barcelona Clinic Liver Cancer

We also used RCS to model the association between age and mortality at 40, 50, 60, 70, and 80 years of age. The hazard ratio (HR) at 45 years of age was defined as 1 based on the patient’s age HR and its 95% confidence interval (CI), and was divided into BCLC stages 0-B and C-D, as shown in Fig. 2. In BCLC stage 0-B, all-cause mortality increased with age, with a rapid increase at around age 60 years (nonlinear, *p* < 0.05). There was a significant U-shaped relationship between age and all-cause mortality in BCLC stage C-D; there was a significant reduction in risk at the lower age range, reaching a minimum risk around age 51 years followed by an increase (nonlinear p < 0.05).

### Fine-gray model of the two causes of death for different BCLC stages

**Fig. 3:**
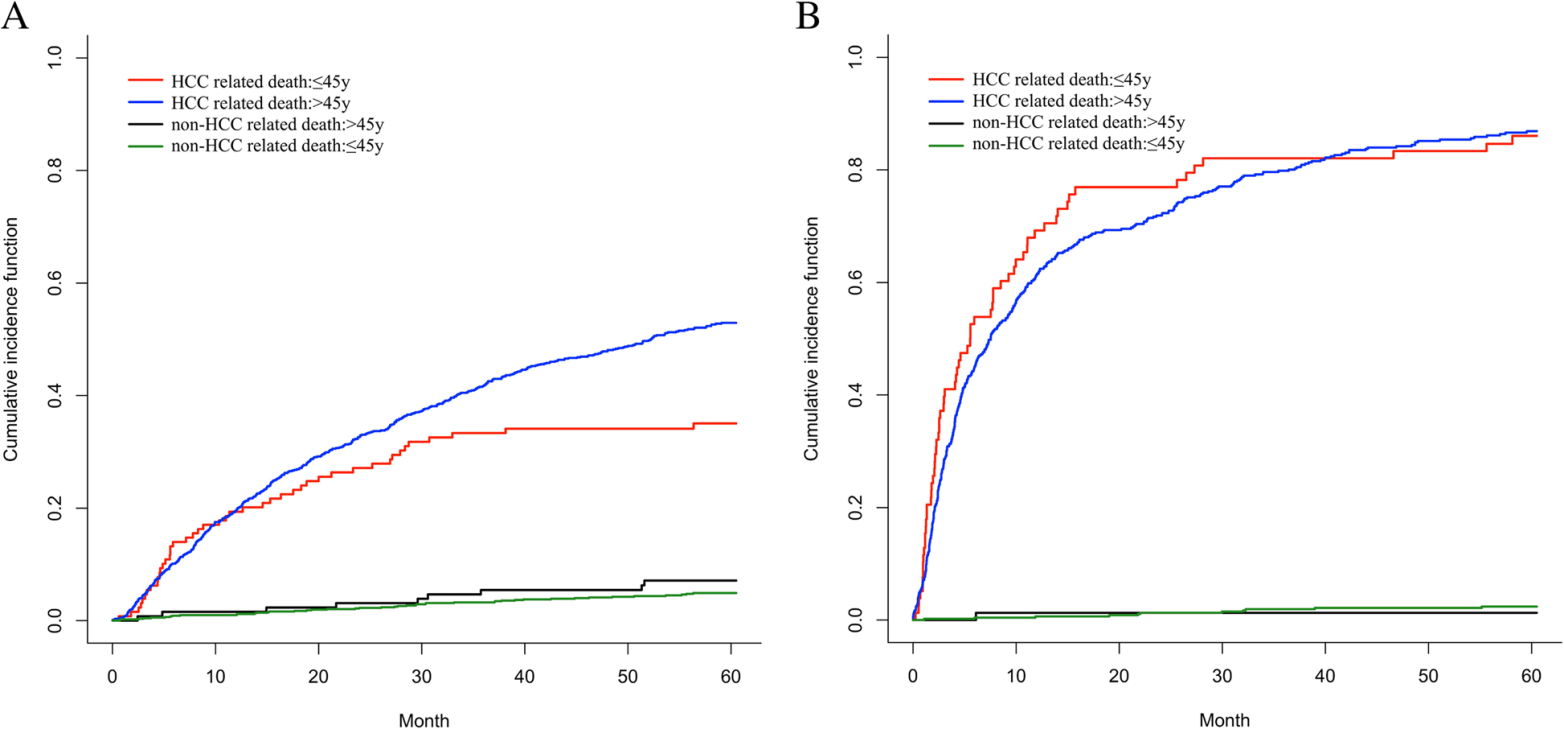
In BCLC 0-B group (**A**) and BCLC C-D group (**B**), overall survival by cause of death was observed in >45y and ≤ 45y age groups. The curves were estimated based on a Fine-Gray model. HCC, hepatocellular carcinoma

The 5-year outcomes of the two causes of death, HCC-related death and non-HCC-related death, significantly differed for BCLC stage 0-B patients aged ≤45 years and older (*p* = 0.0019); non-HCC-related death was not significantly different (*p* = 0.2897). The cumulative incidence functions at month 50 were 52.9, 35.0, 4.9, and 7.1%, respectively, for patients aged ≤45 years and older BCLC stage C-D (*p* = 0.3581, *p* = 0.5462, respectively) and the cumulative incidence functions at month 50 was 86.9, 86.0, 2.4, and 1.3%, respectively (Fig. 3A and B).

### Univariate and multivariate analyses of BCLC stages 0-B

A multivariate Cox proportional risk model was used to analyze the influence of baseline indicators on the 5-year mortality of patients with liver cancer (Table 2). Being younger (HR = 0.74, 95% CI, 0.55–0.98, *p* = 0.037) and having a tumor size ≥5 cm (HR = 2.46, 95% CI, 2.05–2.95, *p* < 0.001) were found to be independent risk factors affecting OS. The independent protective factors of PFS in patients with HCC (Table 2) were younger age (HR = 0.71, 95% CI, 0.55–0.91, *p* = 0.008) and tumor size ≥5 cm (HR = 2.41, 95% CI, 2.04–2.85, p < 0.001). A younger age showed the strongest protective effect after multivariate adjustment.

**Table 2:** Prognostic factor analysis for overall survival and progression-free survival

Variables	Overall survival						Progression-free survival					
	Univariate analysis			Multivariate analysis			Univariate analysis			Multivariate analysis		
	HR	95%CI	*p*.value	HR	95%CI	*p*.value	HR	95%CI	*p*.value	HR	95%CI	*p*.value
Sex[Female]	0.93	0.76–1.1	0.45				0.93	0.78–1.1	0.44			
Age group[≤45y]	0.69	0.52–0.91	0.0093	0.74	0.55–0.98	0.037	0.68	0.53–0.87	0.0023	0.71	0.55–0.91	0.008
Alcohol abuse[alcohol]	1.2	0.98–1.4	0.079				1.1	0.95–1.3	0.21			
Family history of HCC[No]	0.75	0.45–1.3	0.28				0.91	0.59–1.4	0.65			
Tumor size[> 5 cm]	2.4	2–2.9	< 0.001	2.46	2.05–2.95	< 0.001	2.4	2–2.8	< 0.001	2.41	2.04–2.85	< 0.001
PVTT at baseline[Yes]	2	0.82–4.8	0.13				1.3	0.55–3.2	0.53			
WBC (10^9/L) [High>4]	0.97	0.83–1.1	0.75				0.95	0.82–1.1	0.46			
PLT(10^9/L) [High> 100]	0.95	0.81–1.1	0.56				0.99	0.85–1.1	0.89			
ALT(U/L) [High> 50]	1.2	0.96–1.4	0.12				1.2	1–1.4	0.035			

A Cox proportional hazards regression model for OS and PFS was used. The reference category for each categorical variable is in the square brackets in first column. HR, hazard ratio; OS, overall survival; PFS, progression-free survival; PVTT, portal vein tumor thrombus; WBC, white blood cell; PLT, platelet; ALT, alanine aminotransferase

### Subgroup analysis of BCLC stages 0-B

**Fig. 4:**
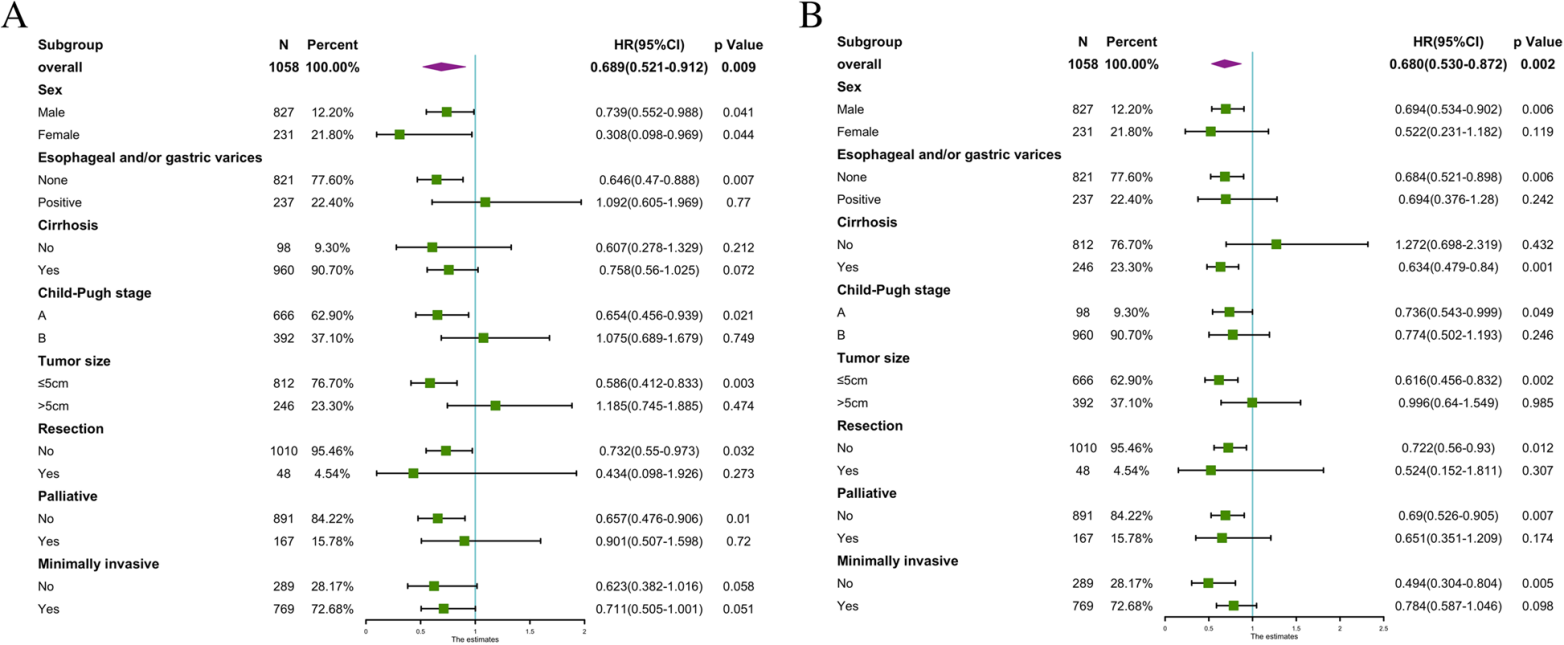
Forest map comparing mortality risk in overall survival (**A**) and progression-free survival (**B**) between BCLC 0-B group HCC patients in > 45y and age ≤ 45y age groups. Numbers in parentheses are 95% CIs. HR, hazard ratios; CI, confidence interval

Subgroup analysis of OS revealed a 26.1% reduction in the risk of death in young men (HR = 0.739; 95% CI, 0.552–0.988) (Fig. 4A). Compared with older patients, the risk of death was 35.4% lower in young men without esophageal or gastric varices (HR = 0.646; 95% CI, 0.47–0.888) and 41.4% lower in young men with tumor size ≤5 cm (HR = 0.586; 95% CI, 0.412–0.833). Subgroup analysis of PFS showed that compared with older patients, young people without esophageal or gastric varices had a 31.6% lower risk of death compared with patients (HR = 0.684; 95% CI, 0.521–0.898) (Fig. 4B). Young people with a tumor size ≤5 cm had a 38.4% lower risk of death (HR = 0.616; 95% CI, 0.456–0.832). Patients with BCLC stage 0-B tumor size ≤5 cm in size had better 5-year OS and PFS compared with patients with tumor size > 5 cm (both *p* < 0.0001; Fig. S1A, B). In contrast, patients with esophageal or gastric varices had a better 5-year OS compared to those without varices (*p* = 0.0019), whereas the PFS was not significantly different between these groups (*p* = 0.2; Fig. S1C, D).

## Discussion

Patient age is an important prognostic factor in various malignancies. In some cancers, the prognosis is poorer in young people, whereas cancers show good survival outcomes in middle-aged patients [13]. Whether age affects the survival of patients with liver cancer is unclear. According to American guidelines for the management of HCC, screening should begin at the age of 40 years in Asian men and 45 in women. Therefore, we evaluated HCC according to the BCLC staging in younger (≤45 years) and older (> 45 years) patients.

Kim et al. [14] reported that younger patients had a worse prognosis compared to older patients, and Furuta et al. [15] found no significant difference in OS between younger patients and older patients with HCC. However, some studies showed that younger patients have better survival rates than older patients [16, 17] or better survival rates when treated with systemic chemotherapy [18]. This is consistent with the results of our study. In survival analysis based on the BCLC staging system, we showed that age affects the long-term survival of patients with BCLC stage 0-B HCC. In BCLC stage 0-B, younger patients with HCC (≤45 years) had better 5-year OS and PFS compared to older patients with HCC (> 45 years).

Our data revealed a higher proportion of cirrhosis and worse Child-Pugh status in the older group, leading to better long-term survival in the younger group (≤45 years) than in the older group (> 45 years). This may be because of the decrease in hepatic mass and hepatic blood flow with aging; additionally, the pharmacokinetics of many drugs change with age [19–21]. Zoli et al. [22] reported that portal venous blood flow rates were significantly lower in older patients than in younger patients. Younger patients have relatively better liver quality, whereas older patients show atrophy due to reduced liver cell counts. Thus, liver function is an important factor affecting prognosis, which is consistent with our findings [23]. Younger patients with HCC may have a higher likelihood of hepatic surrogate function, which is an important factor affecting prognosis [24]. Therefore, a better liver reserve in younger patients with HCC may help prolong survival after 5 years.

We analyzed the relationship between age, mortality, and different causes of death. We used restricted cubic sample bars to flexibly model and visualize the relationship between predicted age and all-cause mortality. In BCLC stage 0-B, age was positively correlated with all-cause mortality and younger patients benefited, which is consistent with our previously observed results. There was a clear U-shaped relationship between age and all-cause mortality in BCLC C-D, with younger patients showing higher mortality rates and a more aggressive phenotype in late HCC compared to older patients [25]. Another study showed that despite having larger tumors and advanced tumor staging, younger patients tend to be treated more aggressively and frequently compared to patients aged 60 years or older, who tend to avoid aggressive treatment [14]. The relationship between the different causes of death and age was analyzed. According to a Japanese study, the risk rate of HCC-related or liver-related death after hepatectomy was almost 1, whereas the risk rate for other causes of death increased significantly with age [26]. However, Motoyama et al. [27] showed that although mortality from other causes is high in the elderly, the 5-year survival rate is lower in the elderly than in younger patients. This is consistent with our findings for the different causes of death in the two age groups of patients with HCC. At BCLC stage 0-B, the cumulative incidence curves of HCC-related deaths significantly differed (*p* < 0.05) (data not shown), with higher death rates in the older group than in the younger group, but the cumulative incidence curves of non-HCC-related deaths were essentially the same. Younger patients may be less likely than older patients to die from liver cancer at stage 0-B BCLC. The disease progresses differently in different stages in younger and older patients, which may help explain why previous studies showed differing results regarding whether age affects prognosis.

One study showed that younger patients typically present late with multiple lung metastases, whereas the disease is more likely to be detected in older patients during routine screening; therefore, the difference in reported clinicopathological features between the two groups may be related to differences in clinical presentation and screening strategies [5]. Another study also showed that young patients with HCC mostly had advanced disease at the time of detection and had larger tumors compared to the older group [15]. This suggests that early detection in younger patients, such as through screening, is beneficial, which is supported by our results.

This study had some limitations. First, this was a retrospective study and was somewhat constrained by differences in clinical characteristics between the two groups, resulting in selection bias. However, the homogeneity of the study population and combined data on risk factors minimized potential confounding factors. Second, this was a single-center study, and further research is needed to better understand the impact of age on the prognosis of liver cancer in other ethnicities or regions. Finally, our study focused on long-term survival outcomes, and additional studies are needed to evaluate short-term survival outcomes.

In conclusion, our study may be useful for predicting the prognosis of younger patients with HCC. In BCLC 0-B stage, age affects the long-term prognosis of patients and is positively correlated with the mortality rate. Young patients with HCC with stage BCLC 0-B would benefit. Therefore, young patients with liver diseases, such as chronic hepatitis B, should undergo screening.

## Supplementary Information

**Additional file 1: Table S1**. Baseline characteristics of study patients after propensity score analysis. **Figure S1**. The Kaplan-Meier survival curves of Overall survival (OS) and Progression-free survival (PFS) in BCLC 0-B group. (A-B) The OS (A) and PFS (B) in tumor size. (C-D) The OS (C) and PFS (D) in esophageal and/or gastric varices. (E-F) The OS (E) and PFS (F) Child staging A and B.

## Data Availability

The datasets used or analyzed during the current study are available from the corresponding author upon reasonable request.
