# Supplementary material for: The survival strength of younger patients in BCLC stage 0-B of hepatocellular carcinoma: basing on competing risk model
Source: BMC Cancer. 2022 Feb 18;22:185. doi: 10.1186/s12885-022-09293-x (PMC8855543; doi:10.1186/s12885-022-09293-x)
Supplement: Supplementary file 1 — Additional file 1: Table S1. Baseline characteristics of study patients after propensity score analysis. Figure S1. The Kaplan-Meier survival curves of Overall survival (OS) and Progression-free survival (PFS) in BCLC 0-B group. (A-B) The OS (A) and PFS (B) in tumor size. (C-D) The OS (C) and PFS (D) in esophageal and/or gastric varices. (E-F) The OS (E) and PFS (F) Child staging A and B. [file 12885_2022_9293_MOESM1_ESM.pdf]

The Survival Strength of Younger Patients in BCLC stage 0-B of Hepatocellular Carcinoma: basing on Competing Risk Model

Huiwen Yan[1,2,*]; Xinhui Wang[1,*]; Xiaoli Liu[1]; Peng Wang[1]; Lihua Yu[1]; Dongdong Zhou[1]; Zhiyun Yang[1]

1 Center of Integrative Medicine, Capital Medical University Affiliated Beijing Ditan Hospital, Beijing 100015, P.R. China

2 Dongzhimen Hospital, Beijing University of Chinese Medicine, Chaoyang District, Beijing 100029, P.R. China

*These authors contributed equally to this work.

Corresponding author: Zhiyun Yang, MD, PhD, Center for Integrative Medicine, Beijing Ditan Hospital, Capital Medical University, No. 8 Jing Shun East Street, Beijing 100015, P.R. China. Tel.: 86-10-84322148, Fax: 86-10-84322148, Email: yangzhiyun2016@163.com.

Table S1. Baseline characteristics of study patients after propensity score analysis.

Demographic and clinical values	Total (N=414)	Age group>45y (N=207)	Age group≤45y (N=207)	P-value
**Patient characteristics**				
**Sex**				1
Male	366 (88.4)	183 (88.4)	183 (88.4)	
Female	48 (11.6)	24 (11.6)	24 (11.6)	
**Alcohol abuse**				0.136
No alcohol	236 (57.0)	110 (53.1)	126 (60.9)	
Alcohol	178 (43.0)	97 (46.9)	81 (39.1)	
**Family history of HCC**				0.057
No	400 (96.6)	204 (98.6)	196 (94.7)	
Yes	14 (3.4)	3 (1.4)	11 (5.3)	
**HBsAg**				0.001
Negative	35 (8.5)	27 (13.0)	8 (3.9)	
Positive	379 (91.5)	180 (87.0)	199 (96.1)	
**HCV**				0.127
Negative	407 (98.3)	201 (97.1)	206 (99.5)	
Positive	7 (1.7)	6 (2.9)	1 (0.5)	
**Esophageal and/or gastric varices**				0.809
No	327 (79.0)	165 (79.7)	162 (78.3)	
Yes	87 (21.0)	42 (20.3)	45 (21.7)	
**Cirrhosis**				0.003
No	46 (11.1)	13 (6.3)	33 (15.9)	
Yes	368 (88.9)	194 (93.7)	174 (84.1)	
**PVTT at baseline**				0.363
No	311 (75.1)	160 (77.3)	151 (72.9)	
Yes	103 (24.9)	47 (22.7)	56 (27.1)	
**Child Staging**				0.02
A	220 (53.1)	96 (46.4)	124 (59.9)	
B	139 (33.6)	81 (39.1)	58 (28.0)	
C	55 (13.3)	30 (14.5)	25 (12.1)	

**Tumor characteristics**				
**Tumor multiplicity**				1
Solitary	247 (59.7)	123 (59.4)	124 (59.9)	
Multiple	167 (40.3)	84 (40.6)	83 (40.1)	
**Tumor size**				1
≤5cm	265 (64.0)	132 (63.8)	133 (64.3)	
>5cm	149 (36.0)	75 (36.2)	74 (35.7)	
**BCLC staging**				1
0-B	258 (62.3)	129 (62.3)	129 (62.3)	
C-D	156 (37.7)	78 (37.7)	78 (37.7)	
**Preoperative laboratory tests**				
**WBC (10^9/L)**				0.115
Low≤4	134 (32.4)	75 (36.2)	59 (28.5)	
High>4	280 (67.6)	132 (63.8)	148 (71.5)	
**PLT(10^9/L)**				0.018
Low≤100	203 (49.0)	114 (55.1)	89 (43.0)	
High>100	211 (51.0)	93 (44.9)	118 (57.0)	
**ALT (U/L)**				0.092
Low≤50	281 (67.9)	149 (72.0)	132 (63.8)	
High>50	133 (32.1)	58 (28.0)	75 (36.2)	
**Type of treatment**				
**Resection**				1
No	390 (94.2)	195 (94.2)	195 (94.2)	
Yes	24 (5.8)	12 (5.8)	12 (5.8)	
**Palliative**				1
No	310 (74.9)	155 (74.9)	155 (74.9)	
Yes	104 (25.1)	52 (25.1)	52 (25.1)	
**Minimally invasive**				1
No	152 (36.7)	76 (36.7)	76 (36.7)	
Yes	262 (63.3)	131 (63.3)	131 (63.3)	

p Value between Age group >45y and Age group ≤45y groups.

PVTT, portal vein tumor thrombus; WBC, white blood cell; PLT, platelet; ALT, alanine aminotransferase.

Fig. S1

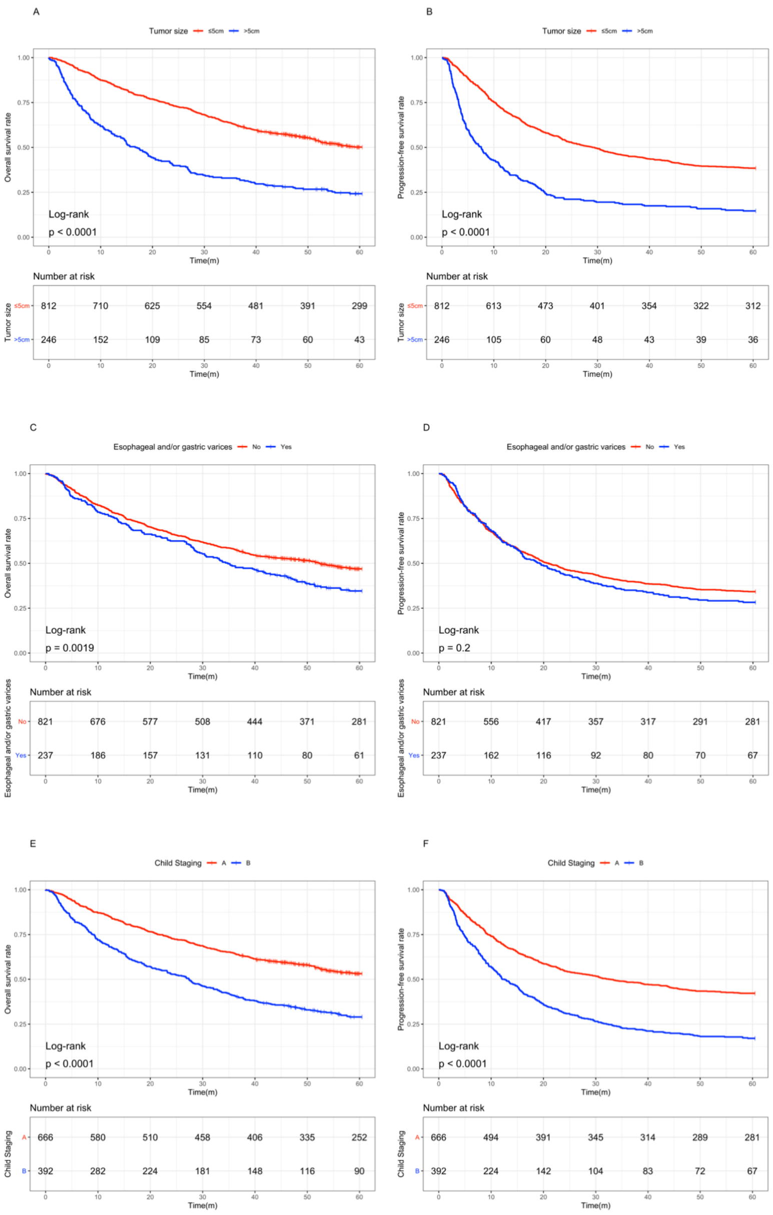

Fig. S1. The Kaplan-Meier survival curves of Overall survival (OS) and Progression-free survival (PFS) in BCLC 0-B group. (A-B) The OS (A) and PFS (B) in tumor size. (C-D) The OS (C) and PFS (D) in esophageal and/or gastric varices. (E-F) The OS (E) and PFS (F) Child staging A and B.
